# Tuberculosis-associated hemophagocytic lymphohistiocytosis with initial presentation of fever of unknown origin in a general hospital: An analysis of 8 clinical cases

**DOI:** 10.1097/MD.0000000000006575

**Published:** 2017-04-21

**Authors:** Yun Zhang, Guangyu Liang, Hongli Qin, Yuanjie Li, Xuejun Zeng

**Affiliations:** Department of General Internal Medicine, Peking Union Medical College Hospital (PUMCH), Chinese Academy of Medical Science (CAMS), and Peking Union Medical College (PUMC), Beijing, China.

**Keywords:** antitubercular therapy, fever of unknown origin, hemophagocytic lymphohistiocytosis, immune therapy, prognosis, tuberculosis

## Abstract

The study aimed to investigate the clinical features and prognoses of patients with tuberculosis (TB) who had secondary hemophagocytic lymphohistiocytosis (HLH).

Patients first presenting with fever of unknown origin, who were ultimately diagnosed with TB-associated secondary HLH, were assessed retrospectively. We summarized and analyzed clinical manifestations, laboratory examinations, diagnoses, treatments, and prognoses of patients using clinical data, outpatient follow-up, and telephone follow-up in combination with literature review.

Among patients admitted to the hospital with fever of unknown origin in the past 10 years, 371 patients were diagnosed with TB. Among them, 8 cases were diagnosed as tuberculosis-associated HLH (TB-HLH). The proportion of females among TB-HLH patients was higher than the proportion of females among TB patients. Within the same time period, 227 cases met the diagnostic criteria for HLH, among which TB-HLH patients accounted for 3.52% of the cases. None of the 8 TB-HLH patients had underlying diseases, and a majority of them had short symptom durations, rapid progression, along with multisystem and multiorgan dysfunctions. Their clinical manifestations were inconsistent with the typical clinical manifestations and imaging results characteristic of TB. Compared with patients with TB in our hospital during the same period, the 8 TB-HLH patients had a higher proportion of blood-disseminated TB and tuberculous meningitis. Apart from this, the hematological damage in these patients was higher than the common clinical manifestations of TB, and they also had a high proportion of respiratory failure. All 8 TB-HLH patients received antitubercular therapy, and 6 of them were also treated for HLH. However, their morbidity and mortality were significantly higher than that for reported cases of TB-HLH cases, both domestically and abroad, which may be attributed to the fever of unknown origin.

Patients with TB-HLH had poor prognoses and no specific clinical manifestations. Therefore, cases of atypical TB and severe TB should be carefully monitored to achieve early diagnosis and early intervention.

## 1 Introduction

The incidence rate of tuberculosis (TB) is very high in China, and data from Centers for Disease Control in recent years have shown a growing number of patients with multidrug-resistant TB and refractory TB. Treatment for patients with severe TB infection has drawn widespread attention.^[1]^ Further, the diagnosis of fever of unknown origin (FUO), which is commonly caused by infectious diseases, autoimmune diseases, and malignant tumors, has become a problem that clinicians often face in large general hospitals. With a diverse range of clinical manifestations, TB is a common cause of FUO.

Hemophagocytic syndrome, also known as hemophagocytic lymphohistiocytosis (HLH), is associated with cytokine storm and inflammation, which seriously impact quality of life. HLH is rare, with a lower incidence in adults than in children, and usually presents secondary to cancer, infections, autoimmune, and other diseases. Among infectious diseases, secondary or acquired HLH is commonly associated with viral infections, such as Epstein-Barr virus and cytomegalovirus, and also bacterial infections; secondary HLH can also be caused by TB-HLH.^[2]^

Hemophagocytic lymphohistiocytosis is a severe complication of TB infection and has a high mortality. For patients with FUO, HLH-related clinical manifestations sometimes present before the final diagnosis of TB. Existing literature on TB-HLH in China and abroad are limited to case reports. Thus, it is necessary to understand the characteristics of secondary TB-HLH and its prognosis to achieve early recognition and treatment.

## 2 Methods

### 2.1 Subjects

Using the medical record system of Peking Union Medical College Hospital (PUMCH), a total of 371 patients who were admitted to our hospital with FUO and finally diagnosed with TB in the past 10 years (from January 2006 to December 2015) were analyzed (including 3 categories: patients with positive results using acid-fast bacilli-associated etiological examination, patients whose histopathological findings were inconsistent with TB, and patients who obtained a good outcome with clinical diagnosis and diagnostic antitubercular therapy [ATT]). Among these, a total of 8 cases were diagnosed as TB-HLH. Within the same period, a total of 294 patients diagnosed with HLH had been discharged from the hospital, and a total of 227 cases were verified to satisfy the HLH-2004 diagnostic criteria with complete information available for analysis.

### 2.2 Diagnostic criteria

We verified patient diagnoses and retrospectively analyzed clinical manifestations, physical signs, laboratory examinations, and treatments. These analyses were completed in combination with literature review and collection of follow-up diagnosis and treatment information for analysis; follow-up was accomplished by means of outpatient contact, telephone, and the use of a mobile phone app. FUO was defined as: fever that lasted ≥3 weeks; temperature >38.3°C ≥2 times; and no diagnosis even after evaluation of complete disease history, physical examination, and routine laboratory tests for ≥1 week.^[3]^

To diagnose HLH, we used the HLH-2004 trial diagnostic criteria. The H-score system was also referred for performing diagnosis, as described in Tables 1–3.^[4,5]^

**Table 1:**
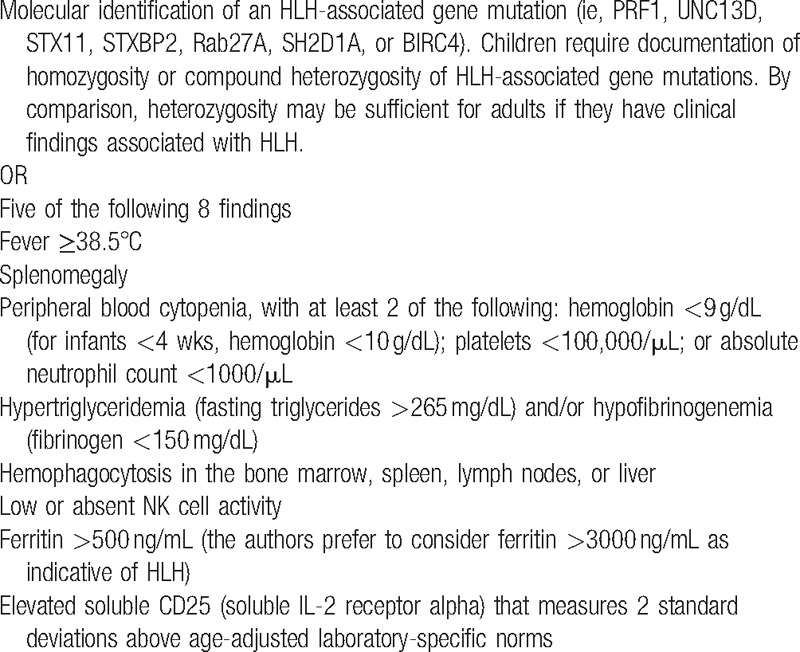
Diagnostic criteria of HLH-2004.

**Table 2:**
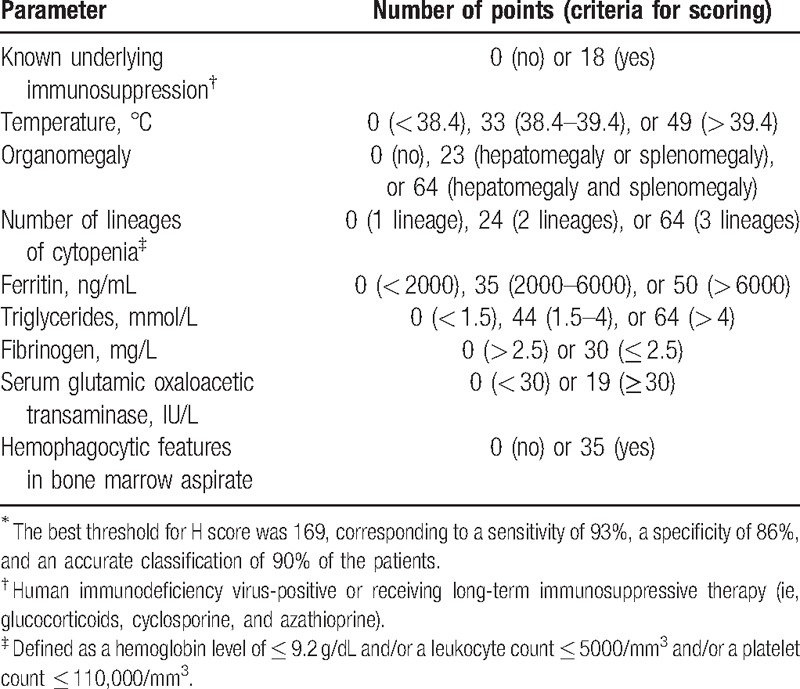
The H score system^∗^.

**Table 3:**
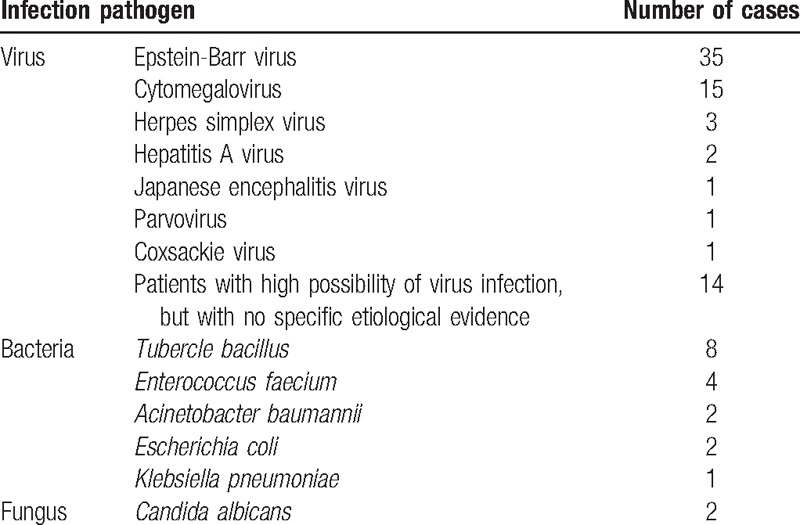
Patients diagnosed with infection-associated HLH at Peking Union Medical College Hospital from 2006 to 2015.

### 2.3 Statistical analysis

SPSS 18.0 (SPSS Inc., Chicago, IL) was used for all statistical analysis. Categorical data are presented as numbers. Statistical comparisons between experimental groups were analyzed using Fisher exact test, and a 2-tailed *P* < 0.05 was considered statistically significant.

### 2.4 Ethics approval and consent to participate

All human and animal studies have been approved by the Ethics Committee of Peking Union Medical College Hospital and have therefore been performed in accordance with the ethical standards specified in the 1964 Declaration of Helsinki and its later amendments. All study participants provided their written informed consent before inclusion in the study.

## 3 Results

### 3.1 Patient characteristics

Among patients admitted to our hospital with FUO, who were finally diagnosed with TB, the ratio of male to female was 1.14:1. The average age was 42.1 ± 17.5 (mean ± standard deviation [SD]) years (range 18–82 years). Among these patients, HLH incidence rate was 2.16%, with male-to-female ratio of 1:3 and an average age of 46.6 ± 19.3 years (range 23–78 years). There was a higher proportion of females among the TB-HLH patients than the proportion of females among TB patients (*P* = 0.001).

Among the 227 HLH patients, there were 91 infection-associated HLH patients. Detailed information is shown in Table 3. In total, 8 patients were diagnosed with TB-HLH, accounting for 3.52% of HLH cases. The 8 patients were identified and screened for other possible causes of HLH during the course of disease evolution, and no positive results were found.

### 3.2 Clinical features and laboratory results

The 8 patients diagnosed with TB-HLH had no underlying diseases (Table 4). Overall, the disease had no specific clinical manifestations or symptoms, and the patients manifested a fever with no specific features, such as irregular fever and continued fever. Half of the patients (4 cases) presented with skin rash, which presented in different forms. The incidence rates of other positive symptoms including lymphadenectasis, splenomegaly, serous effusion, icterus, bleeding tendency, hepatomegaly, nervous system symptoms (including lethargy, delirium), and arthralgia were 87.5%, 75%, 75% (pericardial effusion 37.5%, pleurisy 25%, and ascites and pelvic fluid 12.5%), 50%, 50%, 37.5%, 37.5%, and 25%, respectively.

**Table 4:**
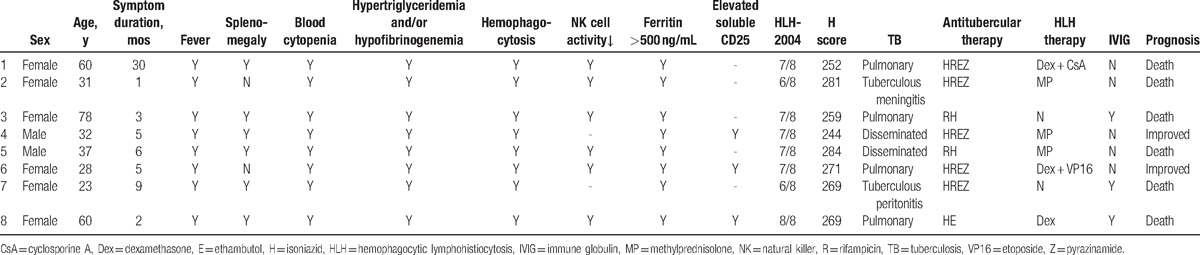
Clinical features of 8 patients with tuberculosis-associated hemophagocytic syndrome.

Regarding laboratory examinations, all patients had abnormalities in liver function, hemogram findings such as moderate and severe anemia, and a reduced number of blood platelets. Other findings associated with blood coagulation included significantly reduced fibrinogen levels, significantly elevated serum ferritin levels, hyperplasia, and histiocytic hemophagocytosis in the bone marrow. Apart from this, the patients showed increased levels of inflammatory markers such as erythrocyte sedimentation rate and C-reactive protein/high-sensitivity C-reactive protein. In addition, 7 patients had a reduced leukocyte or neutrophil counts, 3 of whom exhibited granulocytosis. Six patients showed diminished natural killer (NK) cell activity and reduced numbers of NK cells. Three patients underwent a serum CD25 level examination, and CD25 was elevated in all patients. Four patients underwent serum β2-microglobulin examination, and the level was elevated in 3 patients, whereas it was normal in 1 patient.

All 8 TB-HLH patients underwent necessary infectious disease screening, and all of them presented etiological evidence of TB infection. In 2 cases, the rapid mycobacteria cultures using blood samples showed positive acid-fast stain results. In 1 case, the acid-fast stain of a sputum smear (bronchoscopy) was positive, whereas in 3 cases, positive acid-fast stain sputum using the polymerase chain reaction technique was found (the sputum sample was obtained via bronchoscopy in 1 case). In 1 case, the ascites tested were positive, and in another case, the cerebrospinal fluid was positive, both using the acid-fast stain method. Five of the patients received a T.SPOT TB examination with blood samples, and the results were all 0. For the remaining 3 patients, inspection was not available when they were in the hospital. Moreover, to evaluate the possibility of HLH secondary to cancers, 3 patients underwent positron emission tomography/computed tomography examination, and no clear signs of malignant diseases were found.

Imaging examination revealed varied results: pleural effusion (5 cases), pulmonary infiltrates (4 cases), multiple lung nodules (2 cases), thick-walled cavity (1 case), sign of budding (1 case), ascites (1 case), pericardial effusion (a trace amount, 3 cases), mediastinal lymphadenectasis (4 cases), and retroperitoneal lymphadenectasis (2 cases).

### 3.3 Diagnosis

The HLH-2004 and H-score scoring systems were highly consistent in diagnosing patients with HLH in this study. All 8 TB-HLH patients met the criteria of the 2 diagnostic systems. The mean disease course of all patients diagnosed with HLH was 7.625 months. It is worth noting that HLH was diagnosed before TB in 5 patients. The 8 patients with secondary HLH were diagnosed with pulmonary TB (4 cases), miliary TB (2 cases), abdominal TB (1 cases), and tuberculous meningitis (1 case). Regarding complications, 1 patient suffered a pulmonary bacterial infection during the course of treatment, and another patient was infected with cytomegalovirus after diagnosis (CMV-DNA 1900 copies/mL) that resolved after 1 week with antiviral therapy.

### 3.4 Treatment

Due to rapid development of the disease, 2 patients refused further laboratory tests and treatments, were discharged from PUMCH, and returned home. They did not receive any treatments for HLH (including glucocorticoid and immunosuppressive agents). However, all 8 patients were given ATT (3 patients received treatment only with 2 first-line anti-TB drugs due to severe liver dysfunction, whereas the rest received treatment with 4 first-line anti-TB drugs). Four patients with respiratory failure underwent tracheal intubation and breathed with the help of a ventilator. Two patients experienced septic shock. Six patients received a sufficient amount of glucocorticoid therapy based on the HLH-2004 program, including 1 who received etoposide (VP16) and another who received cyclosporine A (CsA). For supportive treatment, 3 patients received intravenous immunoglobulin therapy, 7 patients received transfusions of red blood cells with fresh frozen plasma, and 5 patients received platelet transfusions for symptomatic therapy.

### 3.5 Prognosis and follow-up

Among the 8 patients with TB-HLH, 3 were admitted to our hospital from the emergency room. All 8 patients were in critical condition during their stay in the hospital. Three patients died during hospitalization, another 3 gave up further treatment and died soon after discharge, 1 patient improved during hospitalization, and 1 improved after receiving treatment in a TB hospital. Currently, the 2 patients who remained relatively stable are no longer receiving ATT (adhere to ATT at least 1 year) or glucocorticoid therapy (adhere to glucocorticoid at least glucocorticoid 8 weeks according to HLH-2004).

## 4 Discussion

China has the second highest incidence rate of TB infections in the world. Therefore, the Chinese government has instituted specific health policies and created specialized TB hospitals to treat confirmed patients with TB. However, TB is known as “a great mimicker” due to its diverse range of clinical manifestations, which make early diagnosis difficult and may even result in misdiagnosis. In large first-class hospitals in China, TB patients are admitted to the hospital mostly due to FUO, and after undergoing abundant and differential diagnosis, they are finally diagnosed with TB. Therefore, the diagnosis of TB that presents as FUO is quite difficult. According to clinical data published by our hospital, TB infection ranks first in the discharge diagnoses of patients admitted with FUO.^[6]^ Meanwhile, in recent years, a growing number of patients have been infected with drug-resistant TB, refractory TB, and severe TB; these patients have to seek medical care in large general hospitals because of difficulties in diagnosis and treatment.^[1]^ TB-HLH is a type of severe TB. Our retrospective case analysis has demonstrated that all TB-HLH patients in our study were admitted to the hospital with FUO, which is undoubtedly a challenge for clinicians. TB-HLH was first reported in the 1980s,^[7]^ and there has been a growing amount of literature on TB-HLH in recent years.^[8,9]^ Nevertheless, these reports both from China and abroad, are single case reports. The current report is the largest single-center case summary in the world thus far.

The pathophysiology of HLH involves excessive immune activation and cytokine storm, but the underlying mechanism of TB-HLH remains unclear, although TB infection disrupts immune homeostasis leading to secondary HLH.^[9]^ In patients with TB, the levels of interferon-γ, tumor necrosis factor-α, and granulocyte/monocyte colony-stimulating factor are higher than those in a healthy population. Moreover, in HLH patients with cytokine storm, the increased levels of cytokines mentioned above along with macrophage colony-stimulating factor and interleukin (IL) family cytokines, including IL-1, IL-6, and IL-18 among others, are important markers. Specifically, *Mycobacterium tuberculosis* could act as an obligate intracellular pathogen after phagocytosis by phagocytic cells to induce TH1-mediated cytotoxicity, activating macrophages and NK cells, further releasing a large quantity of cytokines and chemokines, leading to TB and HLH-related symptoms. Meanwhile, *M tuberculosis* can induce migration of monocytes and macrophages to regional lymph nodes through a mechanism mediated by IL-12 and IL-15, leading to antigen-specific T-cell expansion, stimulation of persistent cytokine release, followed by subsequent activation and proliferation of macrophages.^[10–13]^

According to international literature, the mortality of patients diagnosed with TB-HLH who did not receive ATT is reported to be 100%, whereas ATT in combination with immunotherapy can reduce the mortality rate by 40% to 60%. Due to the long-term characteristics and efficacy of ATT, the utility of sufficient immunotherapy at an early stage of treatment is important to suppress cytokine storm in patients with HLH. Patients who received immunotherapy also experienced significantly higher overall survival rates than patients who did not receive ATT.^[1,9,14–16]^

Meanwhile, a few scholars believe that early and effective ATT is the key to preventing HLH in TB patients, and is the cornerstone of TB-HLH treatment. Increased knowledge of TB infection, early diagnosis, and early treatment can, to a certain extent, prevent the occurrence of HLH.^[9,17,18]^ The mean disease course for patients was 7.6 months in our study. Specifically, there were 6 cases that developed within 6 months of initial presentation, and the disease progressed rapidly before and after admission to our hospital. These patients did not receive ATT previously. Thus, it is inadequate to assess whether effective TB treatment can prevent HLH.

The inclusion criteria were strict in this study. The 8 TB-HLH patients were admitted to the hospital with FUO and with clear evidence of TB. Cases with other secondary HLH were excluded. All of these patients received ATT, and 6 of them received specific treatment for HLH. However, the mortality rate was significantly higher than that for other TB-HLH cases reported domestically and abroad. The reason might be related to their FUO background. In our country, patients diagnosed with TB usually go to specialized TB hospitals for treatment. Our hospital is a center for diagnosis and treatment of rare and tough diseases nationwide. These patients were hospitalized with FUO, representing patients who received more than 3 weeks of treatment before admission with unclear diagnosis, suggesting that this group of patients had atypical clinical manifestations, symptoms, and severe organ damage.

A previous study has shown that, among patients with FUO who were finally diagnosed with TB,^[6]^ those with bloody disseminated TB and tuberculous meningitis accounted for 4% and 6% of the cases, respectively. Most patients with TB and HLH reported previously have had underlying diseases, such as chronic renal failure, cancer, diabetes, acquired immune deficiency syndrome, and renal failure.^[19,20]^ Though this group of patients did not have underlying diseases, they were in poor condition. A high proportion of disseminated pulmonary TB and tuberculous meningitis was also present. In the blood system, the levels of hemoglobin and platelets decreased in the patients with TB-HLH in our study, and coagulation abnormalities were more severe than those commonly observed in patients with TB. Among the 8 patients with TB-HLH, 5 patients eventually developed respiratory failure, and none of them had renal failure.

The typical imaging features of disseminated pulmonary TB include miliary nodule shadows scattered throughout the 2 lungs, and the nodules were usually easy to recognize. However, secondary pulmonary TB typically occurred in the posterior segment of the apical superior lobe and dorsal lower lobe, and it developed more often on the right than on the left. The lung imaging features of this group of patients with disseminated pulmonary TB were atypical, and final diagnoses were based on positive blood cultures. Secondary TB lesions were mainly diffused in both lungs and involved the lower lobe. The predominant morphologies of pulmonary lesions were effusion shadows, nodule shadows, and empty shadows. There was 1 case in our study with diffused interstitial changes in both lungs. In summary, this group of patients had many atypical clinical TB symptoms. Their fever was not low-grade in the late afternoon, which is commonly seen in TB, and the imaging features demonstrated atypical manifestations of TB, presenting rare serious complications mentioned above. These atypical symptoms undoubtedly interfered with the clinicians’ abilities to correctly diagnose TB, but they also helped raise awareness of atypical TB. Cases like these provide a reminder that screening of atypical lesions should not be ignored, such as those in the lower lobe of lungs and those with morphology atypical of TB. Some researchers have suggested that routine tracheal tube brushing or bronchoalveolar lavage fluid inspection for patients suspected to be infected with TB, who have no phlegm or produce negative results in a sputum smear test, can effectively improve the rate of discovery. Yet, for severe TB patients with secondary HLH, low platelet counts and poor coagulation functions might not be able to tolerate invasive inspection and operation for the final diagnosis of TB. Therefore, if suspected, sputum examination and blood culture for TB should be carried out repeatedly, and diagnostic anti-TB treatment should be given actively to those who have no contraindication to ATT.

Most previously reported studies suggest that clinicians consider a diagnosis of HLH when they encounter patients with TB who have reduced levels of blood cells, hepatosplenomegaly, and coagulation abnormalities.^[9,19]^ However, in our study, the majority of HLH manifestations appeared before the diagnosis of TB or acted as a portion of the inflammatory storm that occurred in patients with acute and fulminant TB.

## 5 Conclusions

We noticed an emergence of HLH manifestations in patients with FUO, and thus, the doctors maybe cautioned that screening for TB should be considered during diagnosis. Given the high mortality rate in this group of patients who did not display specific clinical manifestations, careful consideration by clinicians is necessary to achieve early diagnosis, and early intervention may help improve the prognosis.

## Acknowledgments

We thank all of our colleagues at Peking Union Medical College Hospital who have been involved in patient care, medical records management, and research.
